# Identification of new manioc allergens and successful oral immunotherapy in a Brazilian allergic patient

**DOI:** 10.1186/2045-7022-3-S3-P175

**Published:** 2013-07-25

**Authors:** KS Santos, AC Yang, G Gadermaier, CE Galvão, E Vejvar, A Mari, F Ferreira, J Kalil, FFM Castro

**Affiliations:** 1 Department of Medicine, Division of Clinical Immunology and Allergy, University of São Paulo School of Medicine, FMUSP, São Paulo, Brazil; 2 Division of Allergy, Clinics Hospital of FMUSP, São Paulo, Brazil; 3 Christian Doppler Laboratory for Allergy Diagnosis and Therapy, University of Salzburg, Salzburg, Austria; 4 Center for Molecular Allergology, IDI-IRCCS, Rome, Italy; 5 Christian Doppler Laboratory for Allergy Diagnosis and Therapy, University of Salzburg, Salzburg, Austria; 6 Allergy and Immunology, LIM-19, Heart Institute (InCor), São Paulo, Brazil

## Background

Brazil is a tropical country that shows a great biodiversity and is a rich source of new allergens especially from foods. Exotic tropical fruits and plants are highly consumed in the country and due to globalization these products are being exported worldwide. We have recently described the first manioc allergen Man e 5 that cross-reacts with Hev b 5 from latex. There are more than 70 products made up manioc starch such as drugs, soaps and fabrics and manioc allergic patients present from mild to severe reactions.

## Methods

ELISA and ISAC inhibition assays using manioc extract and rMan e 5. Amongst manioc allergic patients we selected a Brazilian woman that lives in North region where manioc is consumed in a daily diet for an oral immunotherapy (OIT) protocol. The patient is a hairdresser, 48 years old, IgE mediated latex allergy presenting cough, sneeze and cutaneous itching. Six years ago she started to have immediate symptoms of urticaria, oral itching and cough after eating different foods prepared with manioc, but more recently she began to present allergic manifestations after inhalatory exposition to manioc starch. Patient was then submitted to OIT with manioc starch solubilized in 10mg/mL. Immunotherapy was divided in two parts: induction and maintenance. In the first phase dose was weekly increased starting with 0.1mL of a 10mg/mL until reaching an amount of 10g of manioc starch. At this point the patient was submitted to oral provocation with 100g of manioc. For maintenance it was indicated daily ingestion of foods prepared with manioc. Clinical evaluation, skin tests and IgE levels after 1 and 6 months were performed.

## Results

Inhibition assays showed that rMan e 5 is not the only manioc allergen and that there is a molecule in manioc cross-reacting with Hev b 6. After OIT patient presented a decrease of the wheal in pricktest (10 to 4mm) and also IgE levels to latex diminished. Oral provocation was negative. Reactions during treatment were mild (abdominal pain and oral itching) and solved spontaneously or using antihistaminic. After two years under maintenance patient keeps asymptomatic eating manioc without restrictions.

## Conclusion

There are at least two manioc allergens cross-reacting with latex. OIT was proved to be safe and efficient in this case allowing free ingestion of manioc. Levels of latex IgE also decreased after treatment. Knowledge of single allergens is important for diagnosis and also to monitor therapeutic success.

## Disclosure of interest

K Santos: None declared, A Yang: None declared, G Gadermaier: None declared, C Galvão: None declared, E Vejvar: None declared, A Mari: None declared, F Ferreira: Grant/research support from Austrian Science Fund (FWF), the Christian Doppler Research Association and Biomay AG, consultant for AllergenOnline Database, Indoor Biotechnologies, and HAL Allergy, J Kalil: None declared, F Castro: None declared.

